# Immunobridging Analysis of Pemivibart for the Treatment of COVID-19: A Therapeutic Gap for the Immune-Compromised Population Remains

**DOI:** 10.1093/ofid/ofag168

**Published:** 2026-04-02

**Authors:** Anna Holmes, Kristin Narayan, Ilker Yalcin, Leijun Hu, Mark A Wingertzahn

**Affiliations:** Invivyd, Inc., New Haven, Connecticut, USA; Invivyd, Inc., New Haven, Connecticut, USA; Invivyd, Inc., New Haven, Connecticut, USA; LH Pharmaceutical Consulting LLC, Carmel, Indiana, USA; Invivyd, Inc., New Haven, Connecticut, USA

**Keywords:** COVID-19, immunocompromised, monoclonal antibody, pemivibart, VYD222

## Abstract

**Background:**

Multiple SARS-CoV-2 receptor-binding domain–directed monoclonal antibodies (mAbs) have demonstrated substantial efficacy for the treatment of COVID-19. However, rapid virus evolution challenges traditional development pathways, as nonsusceptible variants can outpace development and regulatory review. Fortunately, mAb antiviral activity across variants can be measured via clinical serum virus-neutralizing antibody titers that correlate to clinical outcomes from historical mAbs and serve as surrogate biomarkers for efficacy. Analytic immunobridging facilitates rapid assessment of novel mAb efficacy. Immunobridging supported the Emergency Use Authorization of pemivibart, a mAb targeted to the spike protein of SARS-CoV-2 for the prevention of COVID-19 in certain patients with immune compromise. We applied a similar framework to evaluate pemivibart for the treatment of acute COVID-19.

**Methods:**

Complementary methods included the following: (1) strict immunobridging of neutralizing antibody titers of pemivibart to its parent molecule adintrevimab, (2) benchmarking comparison of pemivibart to historical mAbs with demonstrated efficacy in COVID-19 treatment, and (3) dose-response analysis of pemivibart vs comparator mAbs based on a meta-analysis.

**Results:**

Pemivibart demonstrated strict immunobridging to adintrevimab from 4 to >14 days across the variants analyzed. Neutralizing titers of pemivibart were 4- to 12-fold higher than titers for sotrovimab and less than titers for other historical intravenously administered mAbs throughout a 14-day analysis period. Dose-response analysis predicted pemivibart to have equivalent efficacy to all comparators.

**Conclusions:**

Following a similar approach to prevention, immunobridging was demonstrated for pemivibart for the treatment of COVID-19, suggesting substantial antiviral activity. This development methodology provides a roadmap for accelerating novel COVID-19 treatment options amid a changing variant landscape.

## Teaser

We evaluated pemivibart for the treatment of acute COVID-19, aiming to address the urgent unmet needs of patients who are immunocompromised, who remain vulnerable despite vaccination and antiviral therapies. This study utilized an immunobridging approach comparing the neutralizing antibody titers of pemivibart against those of historical monoclonal antibodies with demonstrated efficacy in the treatment of COVID-19 based on multiple complementary methods. In this analysis, pemivibart was predicted to have neutralizing titers in the range of other monoclonal antibodies and comparable efficacy for the treatment of COVID-19. This development methodology, which draws on quantitative virology, biological mechanisms, and the US regulatory response to our initial approach, provides a roadmap for accelerating novel COVID-19 treatment options in the face of a changing variant landscape.

Since the emergence of COVID-19 in 2020, the SARS-CoV-2 virus continues to demonstrate an ability to mutate and evade immunosuppression. Despite the widespread availability of vaccines and antiviral therapies, COVID-19 continues to be one of the leading causes of morbidity and mortality, particularly among individuals who are immunocompromised. In 2023, COVID-19 was the 10th-leading cause of death in the United States [1], and persistent complications associated with postacute sequalae continue to be recognized [2, 3], indicating the critical need for an efficient accelerated path to introduce additional effective treatments.

In humans, antibody responses to SARS-CoV-2, especially high-affinity IgG1 subtypes, emerge days after onset of symptoms and are associated with rapid reduction of viral load once present. High viral load is an independent predictor of acute COVID-19 severity and mortality, making treatment of infection via pharmaceutical monoclonal antibody (mAb) an attractive therapeutic strategy rooted in native human immunobiology.

SARS-CoV-2 receptor-binding domain–directed mAbs have established efficacy in the treatment of COVID-19, demonstrating 51%–87% relative risk reduction in COVID-19–related hospitalization or all-cause death in clinical trials (Table 1) despite different target epitopes, pharmacokinetic profiles, and effector functionalities. While past mAbs proved an effective treatment, their availability was short-lived as emergence of the Omicron variant and associated sublineages rendered them ineffective, resulting in deauthorization by the Food and Drug Administration (FDA). Antivirals are available as treatment options but have limitations, such as drug-drug interactions (nirmatrelvir/ritonavir), renal dosing concerns (remdesivir), marginal clinical efficacy (molnupiravir), and universal lengthy duration of dosing, leaving a critical gap in patient care, especially in high-risk and immunocompromised populations [17–19].

**Table 1.** Half-Maximal Inhibitory Concentrations and Efficacy Against Circulating SARS-CoV-2 Variants of Historical mAbs

mAb	Reported Clinical Efficacy, %^a^	Trial Design Summary	Variants Studied^b^	Pseudovirus IC_50_ (ng/mL) vs Variant	Method
Adintrevimab 300 mg IM	66	STAMP enrolled high-risk unvaccinated individuals 1:1 ADI:PCB [4].	Delta	3.5 vs Delta	Sponsor-reported Monogram pseudovirus assay data to dominant variant in STAMP study [4].
Sotrovimab 500 mg IV	79	COMET-ICE enrolled high-risk unvaccinated individuals 1:1 SOT:PCB [5].	Alpha, Beta, others	70 vs WT	Estimate derived from sponsor-reported pseudovirus assay data in CDER report. WT value noted as 16-fold less than Omicron B.1.1.529/BA.2 IC_50_ of 7.6 nM; no change in susceptibility to Alpha or Beta [6].
Bamlanivimab 700 mg IV	83	BLAZE-1 randomized participants 2:3 BAM:PCB. A subset (44%) was high risk [7, 8].	Beta, others	6.1 vs WT	Average HIV/lentivirus pseudovirus assay data reported [9].
Bamlanivimab/etesevimab 2100 mg IV	87	BLAZE-1 enrolled high-risk unvaccinated individuals with no prior infection 2:3 BAM/ETE:PCB [10].	Alpha, Beta, others	6.9 vs WT	Average HIV/lentivirus pseudovirus assay data reported [9]; no change in susceptibility to D614G or Alpha, reduced susceptibility to Beta.
Casirivimab/imdevimab 1200 mg IV	70	COV-2067 enrolled high-risk unvaccinated individuals with no prior infection 1:1 CAS/IMDE:PCB [11].	Alpha, Beta, others	4.2 vs WT	Average HIV/lentivirus pseudovirus assay data reported [9]; consistent with sponsor-reported VSV pseudotyped virus assay data [12]. No change in susceptibility to Alpha and Beta.
Cilgavimab/tixagevimab >600 mg IM	50.5	TACKLE enrolled a general population with a subset of high-risk unvaccinated individuals 1:1 CIL/TIX:PCB [13].	Alpha, Gamma, Delta	2.1 vs Alpha	Sponsor-reported Monogram pseudovirus assay data to dominant variant in TACKLE study [14].
Bebtelovimab 175 mg IV	NR	BLAZE-4 enrolled low-risk unvaccinated individuals 1:1:1 BEB:BEB/BAM/ETE: PCB or high risk individuals 2:1 to BEB:BEB/BAM/ETE (open label) [15].	Alpha, Delta	2.0 vs WT	Sponsor-reported pseudovirus data reported in CDER report; no change in susceptibility to Alpha and Delta [16].

Abbreviations: ADI, adintrevimab; BAM/ETE, bamlanivimab/etesevimab; BEB, bebtelovimab; CDER, Center for Drug Evaluation and Research; CIL/TIX, cilgavimab/tixagevimab; IC_50_, half-maximal inhibitory concentration; IM, intramuscular; IV, intravenous; mAb, monoclonal antibody; NR, not reported; PCB, placebo; SOT, sotrovimab; VSV, vesicular stomatitis virus; WT, wild type.

^a^Efficacy presented as relative risk reduction in COVID-19–related hospitalization or all-cause death.

^b^Includes the predominant variants in circulation at the time when the study was conducted.

Rapid virus evolution challenges traditional development pathways, as nonsusceptible variants can emerge faster than development and regulatory review of mAbs can occur. Fortunately, candidate mAb antiviral activity across viral variants can be measured and compared via clinical serum virus-neutralizing antibody titers that correlate to clinical outcomes from predicate mAbs, creating a surrogate marker and analytic immunobridging approach useful for rapid assessment of novel mAb efficacy, especially for mAbs engineered from a clinically established molecular ancestor.

Immunobridging is a powerful tool for advancing timely development of treatments for infectious diseases and has been widely used in the development of vaccines to enable rapid vaccine composition evolution and regulatory review. In the context of emerging viral threats with fluctuating transmission dynamics such as COVID-19, immunobridging offers a scientifically grounded and efficient alternative to large-scale efficacy trials that could be routinely deployed to accelerate mAb development, while describing the pharmaceutical amplification of the canonical native human antibody response to acute SARS-CoV-2 infection. It enables assessment of treatment performance across different populations (eg, children, individuals who are immunocompromised) without awaiting natural disease exposure. When supported by strong mechanistic evidence and clinical validation, immunobridging can provide a strong foundation for accelerated development, regulatory decision making, and ultimately more timely patient access to potentially lifesaving interventions [20].

Pemivibart, a potent SARS-CoV-2 receptor-binding domain–directed mAb, offers a rational solution for patients with unmet need. Pemivibart was engineered from its parent molecule adintrevimab with a difference of only 8 amino acids in the Fab. Pemivibart and adintrevimab share identical Fc regions and have demonstrated Fc effector functions, including in vitro antibody-dependent cellular cytotoxicity, cellular phagocytosis, and complement deposition [21, 22]. Adintrevimab was an investigational mAb developed from an antibody isolated from memory B cells of a survivor of the 2003 SARS outbreak and demonstrated efficacy in the treatment of high-risk ambulatory cases of COVID-19 (STAMP trial) and pre- and postexposure prophylaxis of COVID-19 (EVADE trial) against the Delta variant [4, 23]. In the STAMP trial (NCT04805671), a single 300-mg dose of adintrevimab was administered intramuscularly to participants with at least 1 risk factor for severe disease who were diagnosed with COVID-19. The proportion of participants with COVID-19–related hospitalization or all-cause death (primary efficacy endpoint) was 4.7% (8/169) in the adintrevimab arm as compared with 13.8% (23/167) in the placebo arm, a 66% relative risk reduction (*P* = .0047) [4]. Adintrevimab lost activity upon emergence of the Omicron BA.2 variant, and development was suspended.

Pemivibart demonstrates broad and robust neutralizing activity against historical and emerging SARS-CoV-2 variants, including all Omicron sublineages evaluated by Invivyd through September 2025 [21]. On 22 March 2024, the FDA granted Emergency Use Authorization (EUA) for pemivibart for pre-exposure prophylaxis of COVID-19 in adults and adolescents who are moderately to severely immunocompromised, based on immunobridging data from the phase 3 CANOPY trial [21, 24, 25]. In this trial (NCT06039449), participants who were immunocompromised received 4500 mg of intravenous (IV) pemivibart on day 1 and month 3, with surrogate efficacy based on neutralizing antibody titers [24]. Clinical efficacy of pemivibart was later shown in the CANOPY trial in a nonimmunocompromised placebo-controlled cohort, with relative risk reduction in symptomatic COVID-19 of 84% through 6 months, validating immunobridging as a reliable predictor of mAb effectiveness [25].

We therefore applied a similar immunobridging framework to evaluate pemivibart in the treatment of acute COVID-19 in an effort to accelerate treatment availability for patients with an urgent unmet need who remain vulnerable despite vaccination and antiviral therapies. Although we failed to secure FDA authorization of pemivibart for treatment of COVID-19 using this approach, we provide here a roadmap for advancing antiviral treatment via mAb therapy for SARS-CoV-2 globally and possibly for additional viral or microbial diseases in the future.

## METHODS

For treatment of COVID-19, the current study applied an immunobridging approach for pemivibart against contemporary variants, with safety data from the previously described CANOPY clinical trial [24, 25]. The immunobridging analysis used 3 complementary methods:

- Method 1 employed a comparison of calculated neutralizing antibody titers for pemivibart vs adintrevimab based on population pharmacokinetics (popPK) modeling.
- Method 2 compared calculated neutralizing antibody titers of pemivibart vs other SARS-CoV-2–directed mAbs historically used for treatment (but no longer effective) based on popPK modeling.
- Method 3 compared the clinical efficacy and neutralizing dose of comparator mAbs vs pemivibart based on a published meta-analysis [26].

Selected relevant variants evaluated for pemivibart immunobridging include the JN.1-lineage variants JN.1, KP.3.1.1, XEC, and LP.8.1. These 4 variants encompass the range of available half-maximal inhibitory concentration (IC_50_) values against the most prevalent variants in circulation since 2024 [27] (Supplementary Table 1). Although no longer in circulation, JN.1 was the primary variant circulating during the CANOPY trial at the time of the pemivibart EUA application for COVID-19 prevention; KP.3.1.1 and XEC were dominant variants circulating during the CANOPY trial; and LP.8.1 was a dominant variant per the Centers for Disease Control and Prevention’s variant tracker in the spring of 2025 and served as the sequence of the updated 2025 COVID-19 vaccine. Evaluation of the LP.8.1 variant was not included in the EUA application for treatment (not available at the time of submission).

In immunobridging method 1, an immunobridging analysis was employed comparing the neutralizing antibody titers of pemivibart vs adintrevimab. To account for the difference in route of administration for pemivibart (IV) vs adintrevimab (intramuscular), this analysis used geometric mean titers (GMTs) calculated as estimated average drug concentrations from dosing to specified times of interest (area under the curve / time obtained with popPK analysis) divided by pseudovirus neutralization assay IC_50_ against a relevant variant. Adintrevimab titer data were obtained from a phase 1 trial and the STAMP trial during the Delta variant period and compared with pemivibart titers against selected current variants at early timepoints following onset of symptoms. Strict immunobridging is established if the lower bound of the 90% CI of the estimated geometric mean ratio of GMTs from pemivibart compared with adintrevimab is ≥0.8.

In the STAMP trial, the most significant treatment effects were observed if dosing occurred in the first 3 days after symptom onset and the largest impact to viral load was achieved by day 5, suggesting that immunobridging through 5 days is a reasonable target for predicting efficacy [4]. Although mechanisms of actions may differ, sustained antiviral activity through a maximum 5 days is also the intended target for several small molecule products for the treatment of COVID-19 (ie, nirmatrelvir/ritonavir, remdesivir, molnupiravir) [17–19].

In immunobridging method 2, GMTs based on estimated average concentration (area under the curve / time) of pemivibart against relevant variants following a single IV dose were compared with the GMTs of other mAbs previously shown to be efficacious for the treatment of COVID-19 against variants circulating at the time when trials were conducted. Briefly, a pemivibart popPK model and publicly available popPK models of comparator anti–COVID-19 mAbs were used to simulate individual antibody concentration vs time profiles in >2400 virtual subjects based on demographic information from an adintrevimab phase 3 study [14, 28–31]. The geometric mean of the average concentration profile of each antibody was then calculated and derived to obtain GMTs, where IC_50_ values for comparator mAbs were derived from sponsor-reported data or alternative sources using a pseudovirus neutralization assay (Table 1) [6, 9, 12, 14, 16].

Immunobridging method 3 compared the clinical efficacy and neutralizing dose of comparator mAbs vs pemivibart. The dose-response relationship between neutralizing dose and efficacy developed by Stadler et al provides a direct quantitative means of assessing potential therapeutic efficacy based on in vitro neutralization data and mAb dose [26].

## RESULTS

### Immunobridging Method 1

**Figure 1.**
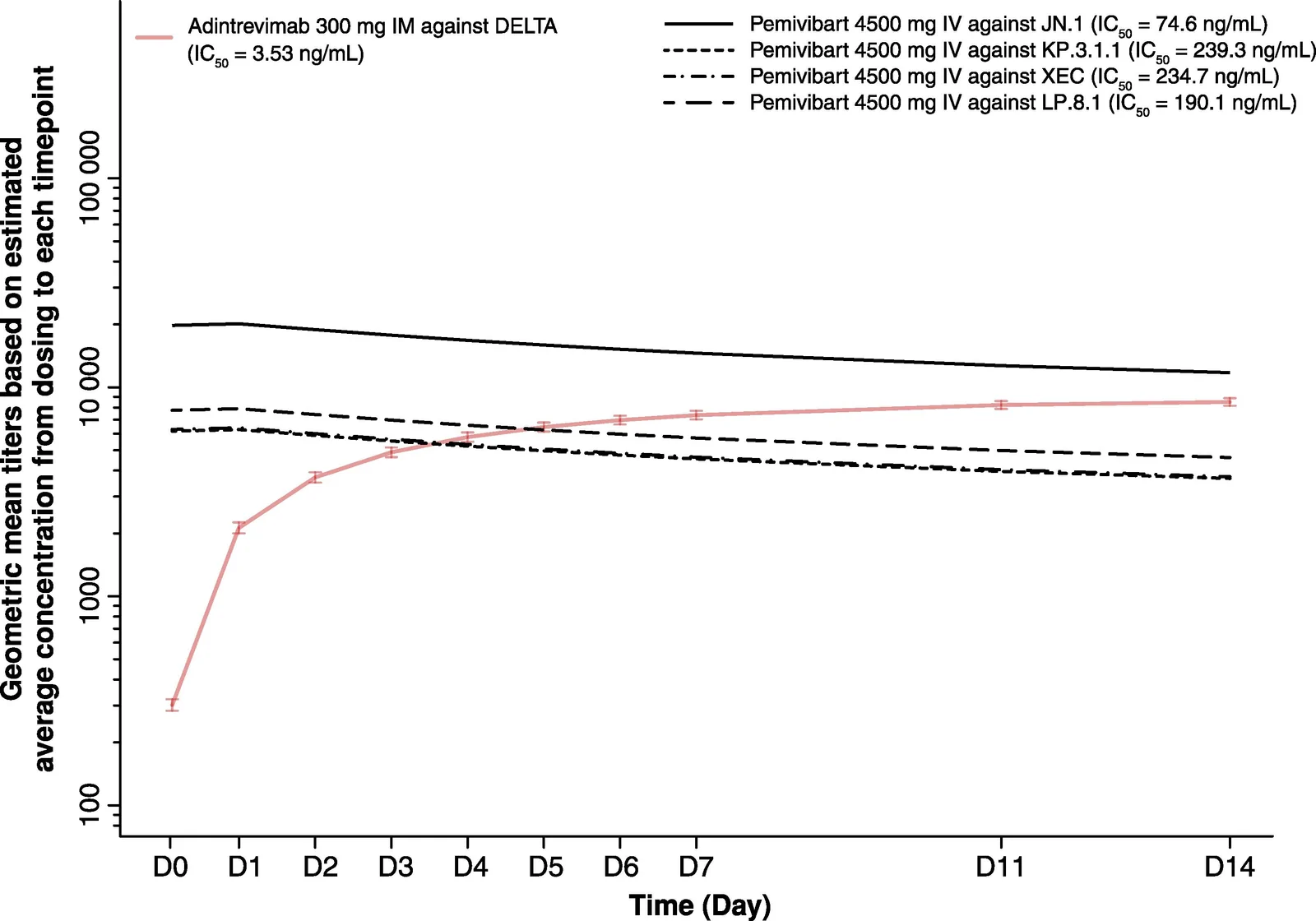
Pemivibart treatment immunobridging to adintrevimab: method 1. Geometric mean titer and 90% CI at each time point for pemivibart and adintrevimab were summarized from population pharmacokinetics model−estimated post hoc concentrations of individuals in the correspondent pemivibart phase 3 study (VYD222−PREV−001) and adintrevimab phase 3 study (ADG20−TRMT−001), respectively. Concentrations were then divided by correspondent IC_50_ values of dominant circulating variants when trials were conducted as described in Supplementary Table 1 for pemivibart and Table 1 for adintrevimab. IC_50_, half-maximal inhibitory concentration; IM, intramuscular; IV, intravenous.

Immunobridging from pemivibart to adintrevimab against the Delta variant was nominally established for selected circulating variants: through 4 days for variants KP.3.1.1 and XEC, through 6 days for LP.8.1, and through >14 days for JN.1 (Figure 1, Table 2). For KP.3.1.1 and XEC, pemivibart GMTs remained at 77% and 79% of adintrevimab GMTs at the immunobridging target of 5 days, respectively. Average titers against all selected variants remained high and stable through 14 days (range, 3662–11 748), suggesting that pemivibart likely continues to provide long-acting antiviral benefit well beyond the acute infection phase, although absolute titers fluctuate depending on the variant analyzed.

**Table 2.** 

	GMR: Pemivibart/Adintrevimab (90% CI)			
Days	JN.1	KP.3.1.1	XEC	LP.8.1
0–1	9.47 (9.04–9.92)	2.95 (2.82–3.09)	3.01 (2.87–3.15)	3.72 (3.55–3.89)
0–2	5.09 (4.87–5.32)	1.59 (1.52–1.66)	1.62 (1.55–1.69)	2.00 (1.91–2.09)
0–3	3.63 (3.48–3.79)	1.13 (1.09–1.18)	1.15 (1.11–1.20)	1.43 (1.37–1.49)
0–4	2.90 (2.79–3.02)	0.91 (.87–.94)	0.92 (.89–.96)	1.14 (1.09–1.19)
**0–5**	**2.47** (**2.38–2.57)**	**0.77** (**.74–.80)**	**0.79** (**.76–.82)**	**0.97** (**.93–1.01)**
0–6	2.18 (2.10–2.27)	0.68 (.66–.71)	0.69 (.67–.72)	0.86 (.82–.89)
0–7	1.98 (1.91–2.05)	0.62 (.59–.64)	0.63 (.61–.65)	0.78 (.75–.81)

Bold indicates targeted immunobridging period of 5 days post-treatment.

Abbreviations: AUC, area under the concentration curve; GMR, geometric mean ratio; PK, pharmacokinetics.

### Immunobridging Method 2

**Figure 2.**
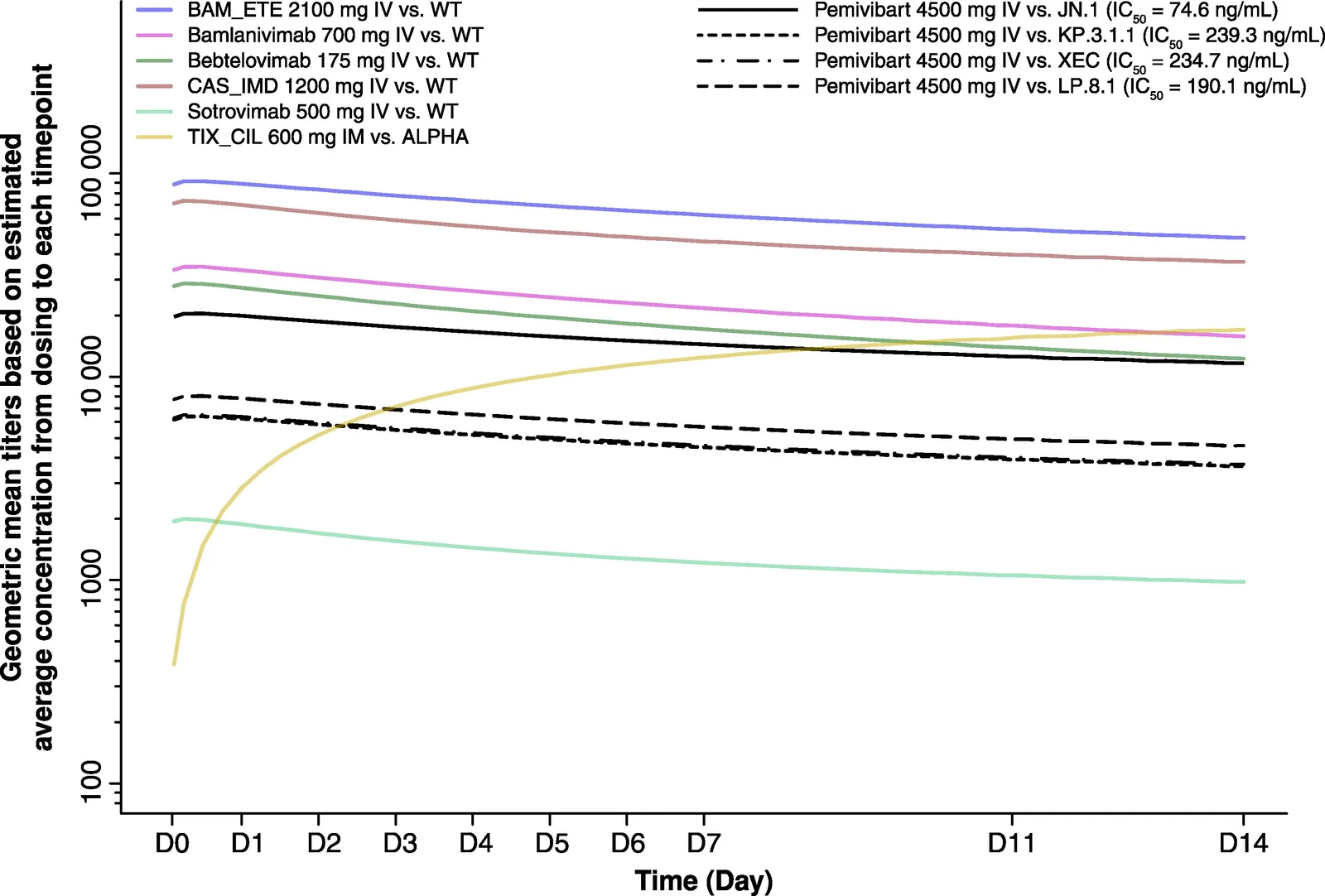
Pemivibart treatment immunobridging to comparator monoclonal antibodies: method 2. The PK profiles for comparator monoclonal antibodies were simulated by using population PK parameters available in the public domain. PK profiles for pemivibart are based on the pemivibart population PK model. Titers were calculated according to published IC_50_ data summarized in Table 1. BAM_ETE, bamlanivimab and etesevimab; CAS_IMD, casirivimab and imdevimab; IC_50_, half-maximal inhibitory concentration; IM, intramuscular; IV, intravenous; PK, pharmacokinetics; TIX_CIL, tixagevimab and cilgavimab; WT, wild type virus.

Pemivibart GMTs to selected variants are between those of sotrovimab and bebtelovimab, with neutralizing titers approximately 4- to 12-fold higher than those provided by a single 500-mg IV dose of sotrovimab through 14 days postdose (Figure 2). Sotrovimab provided clinically meaningful efficacy for the treatment of COVID-19 with a 79% reduction in COVID-19–related hospitalization or all-cause death (Table 1) [5].

### Immunobridging Method 3

**Figure 3.**
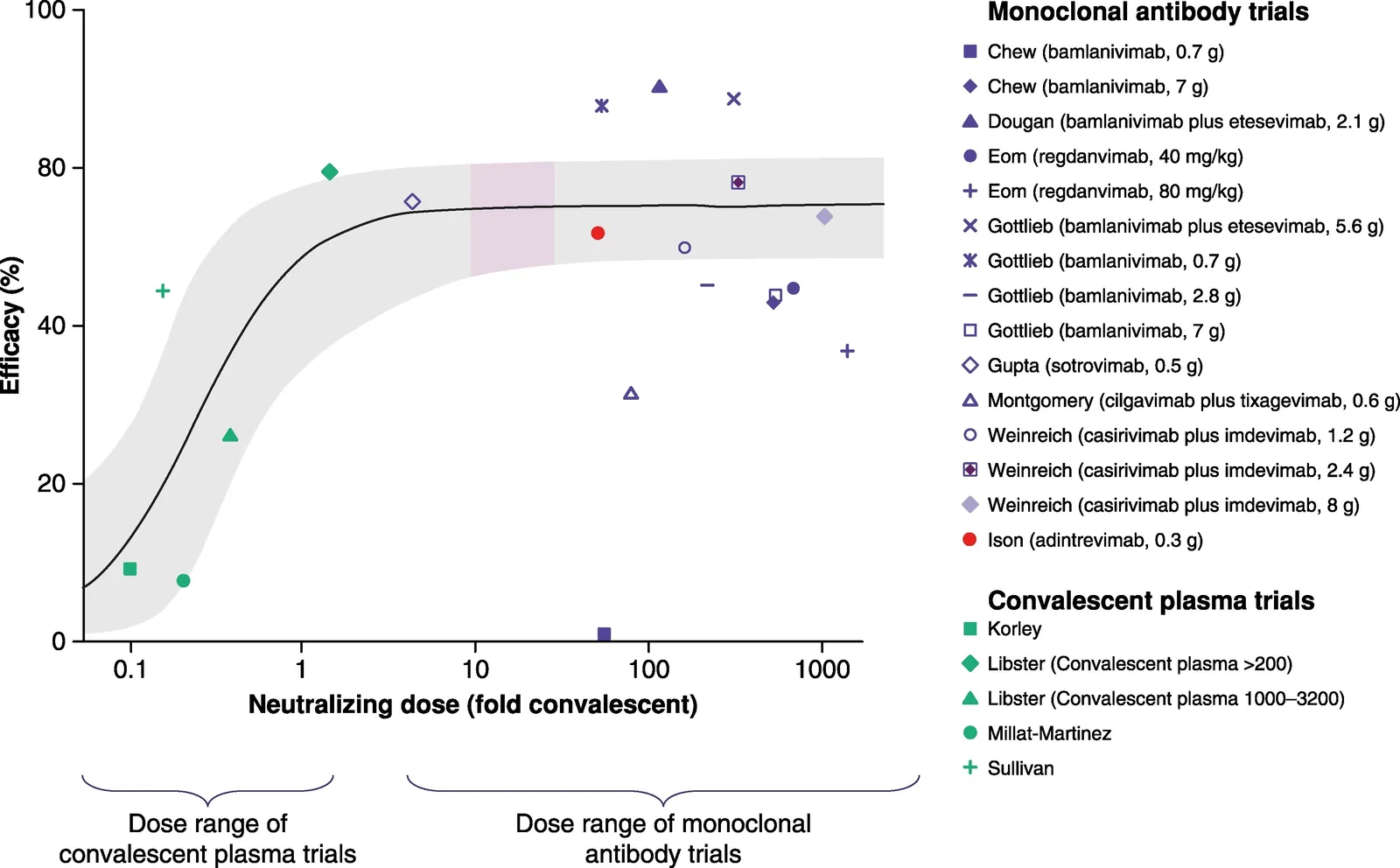
Pemivibart treatment immunobridging meta-analysis: method 3. A logistic model was fitted to the data on efficacy and corresponding neutralizing dose for each study as described by Stadler et al [26]. The fitted curve allows for maximum efficacy (estimated 67.5%). The gray shaded area indicates the 95% CI. The pink shaded area represents the range of neutralizing titers (∼11.2–35.8) against SARS-CoV-2 variants JN.1, KP.3.1.1, XEC, and LP.8.1 following a single dose of pemivibart (4500 mg, intravenous). The red bullet represents adintrevimab neutralizing dose (50.9) based on the half-maximal inhibitory concentration against JN.1 pseudovirus. Figure adapted from Stadler et al [26].

The range of neutralizing doses observed across the specific doses of mAbs with prior EUA for the treatment of COVID-19 is between 4.4 (sotrovimab, 500 mg) and 123.8 (bamlanivimab/etesevimab, 2.1 g; Figure 3). Upon evaluation of pemivibart (4500 mg, IV), the neutralizing titers against selected SARS-CoV-2 variants were 11.2 to 35.8, which falls on the plateau of the efficacy curve. The neutralizing dose of pemivibart is at least 2.5 times greater than sotrovimab for the least susceptible variants, KP.3.1.1 and XEC, and above the range observed across trials of convalescent plasma (0.1–1.5), which is authorized for emergency use for the treatment of COVID-19 in individuals with immune compromise.

It is important to note that based on the meta-analysis from Stadler et al [26], the efficacy rate of all mAbs plateaus once a neutralizing dose is reached. Adintrevimab, with other mAbs authorized for treatment, falls on the plateau of the efficacy curve. These mAbs have significantly higher neutralizing doses than sotrovimab but without increased efficacy. By comparison, 500-mg IV sotrovimab is at the threshold of plateaued maximal efficacy among the comparators against correspondent dominant virus variants. Hence, the clinical equivalence of neutralizing titers of pemivibart to other mAbs is established in this model, where an effective neutralizing dose is present on the plateau of the dose-response curve.

## DISCUSSION

Immunobridging has been a commonly accepted pathway to facilitate approval of vaccines. Pemivibart was the first SARS-CoV-2 mAb to effectively utilize the immunobridging approach to obtain EUA for prevention of COVID-19 prior to the availability of clinical efficacy data [24]. In the prevention immunobridging analysis, strict immunobridging (lower bound of 90% CI ≥0.8) of pemivibart to adintrevimab through 3 months in an immunocompromised population was not met against the JN.1 variant dominant at the time of the analysis. However, the neutralizing titer levels of pemivibart were in line with previous mAbs with established efficacy in the prevention setting, leading to authorization based on the totality of evidence and demonstrating the value of using multiple methods for immunobridging [24].

Since then, pemivibart has shown robust clinical efficacy in the prevention of symptomatic COVID-19 (reverse transcription–polymerase chain reaction confirmed) in an immunocompetent placebo-controlled population through 3 and 6 months, with standardized relative risk reductions of 94% (*P* = .009) and 84% (*P* < .001), respectively, following 2 doses of pemivibart at a 3-month interval in the CANOPY trial. Pemivibart also continued to show clinical efficacy (standardized relative risk reduction, 74%; *P* < .001) against relevant circulating variants through 12 months, despite having waning titer levels, suggesting that while immunobridging is a useful tool for predicting protection, immunobridging target titers do not necessarily define the lower bound at which clinical efficacy is actually present [25].

A similar immunobridging approach was taken to support the use of pemivibart in the treatment of COVID-19. By using neutralizing data against recent dominant variants, pemivibart demonstrated immunobridging through 3 methods, all of which revealed antiviral activity in acute COVID-19 that would support the native antibody response. Strict immunobridging to adintrevimab was achieved through the first 4 days of the treatment period for XEC and KP.3.1.1, through 6 days for LP.8.1, and through >14 days for JN.1 (Figure 1). A period of 5 days from symptom onset has been shown to be most critical in COVID-19 treatment, given clear observations that early intervention and virologic reductions within the first 5 days are correlated with clinical efficacy [13, 32, 33]. Data from the adintrevimab treatment trial (STAMP) showed that an intramuscular injection of adintrevimab, despite failing to generate maximal titer levels until days 7 to 10, correlated to a 66% reduction in hospitalization or death, with the greatest impact on viral load reduction observed at day 5 posttreatment [4]. The pharmacokinetic profile of IV pemivibart lends itself to providing immediate and superior titer levels as compared with adintrevimab for the first 3 days after dosing and comparable titer levels through at least 5 days (Figure 1, Table 2), supporting meaningful clinical potential. In addition, a treatment period of 5 days is targeted for several small molecule products for the treatment of COVID-19. Achieving maximal titers immediately after IV treatment has likely advantages of abolishing clinically significant viremia more quickly and warding against symptom breakthrough or untoward COVID-19 sequalae than other routes of administration.

In comparing pemivibart against other mAbs with known clinical efficacy, neutralizing titer levels of pemivibart most notably remained above the titers of sotrovimab for the full analysis period for all variants tested (>14 days; Figure 2). Likewise, the neutralizing dose of pemivibart falls on the plateau of the dose-response curve generated by Stadler et al [26], establishing clinical equivalence of pemivibart to other mAbs in this model (Figure 3). The totality of the data immunobridging pemivibart to adintrevimab and other comparator mAbs for COVID-19 treatment is similar to that leading to EUA for pemivibart in COVID-19 prevention, with approximately 9 months of safety data from CANOPY and postmarketing safety data also available. Namely, the safety profile of pemivibart, as supported by the CANOPY phase 3 trial, indicates manageable risks, with infusion-related reactions (6%) and rare anaphylaxis (0.6%) across immune-compromised and immune-competent populations mitigated by standard monitoring [21]. Hypersensitivity reactions were also reported during or after use in real-world settings of other approved COVID-19 therapies, such as remdesivir and the nirmatrelvir/ritonavir combination [17, 34]. Therefore, the risk-benefit of pemivibart for the most at-risk populations with unmet need would support its use in COVID-19 treatment.

The results of the analysis were submitted to the FDA for EUA of pemivibart for the treatment of mild to moderate COVID-19. The agency indicated uncertainties in the analysis and was therefore unable to conclude a favorable benefit-risk profile, despite the consistent results from the multipronged immunobridging methodologies. Specifically, minimum titers required for successful treatment and the duration of time that titers must be present in the immune-compromised population have not been defined due to a scarcity of placebo-controlled trials in this high-risk group. In the CANOPY study, neutralizing titers calculated from serum pemivibart concentrations in immunocompromised cases were similar to immunocompetent ones; however, all prior mAb EUAs for treatment were based on studies in immunocompetent cohorts. Clinical efficacy with pemivibart in prevention has demonstrated that antiviral activity is present at much lower titers than those evaluated in any studies with historical mAbs and may apply to treatment, as viral neutralization is a key mechanism of action for both indications. The duration of neutralization required in the immune-compromised population may be longer due to a lack of viral clearance by a faulty or suppressed immune system and may be more susceptible to prolonged shedding and viral mutations, although studies have clearly shown the critical role of early intervention and early viral load reduction in preventing disease progression. Placebo-controlled studies in the immune-compromised population that demonstrate true titer thresholds of clinical effectiveness and duration for treatment are the most straightforward way to definitively answer this question.

Another uncertainty raised by the FDA suggested that immunobridging to comparator mAbs with variability in Fc effector function may contribute to efficacy beyond viral neutralization and therefore may represent an inconsistency in our approach. Pemivibart has intact Fc effector functions, similar to most other historical mAbs for the treatment of COVID-19, with the exception of tixagevimab/cilgavimab and etesevimab [35]. Although overall efficacy conclusions may not be overtly affected, the most appropriate direct immunobridging comparison may be to mAbs developed through the same platform and structural backbone (eg, pemivibart to adintrevimab with only 8 amino acid differences in the Fab and with identical Fc regions). Given that both mAbs have demonstrated similar Fc effector functions, it is a reasonable assumption that the Fc effector function is comparable between pemivibart and adintrevimab when similar GMTs are achieved.

Of note, adintrevimab was particularly potent against the Delta variant, producing titers that were several-fold higher than those expected to produce maximal efficacy. This was recognized as a possible limitation for immunobridging to adintrevimab, as the pemivibart titers required for successful immunobridging were also likely inflated above those required for maximal efficacy. Importantly, however, the dose-response immunobridging analysis suggests that all mAbs with a neutralizing dose equal or greater to sotrovimab should have generally equivalent efficacy, as the maximal efficacy threshold has been reached, which may diminish the importance of showing that neutralizing titers reach a specific numeric level independently or in comparison with other historical mAbs. Here, the totality of evidence seems to favor a reasonable expectation of the effect of pemivibart in the treatment of COVID-19. Patients who are immunocompromised face unique challenges upon SARS-CoV-2 exposure. Current vaccines are insufficient to protect such individuals from infection, resulting in high vulnerability to severe outcomes [34, 36–39]. Indeed, studies report that COVID-19 hospitalization, intensive care unit admission, and mortality rates were approximately 6- to 11-fold higher in immunocompromised populations [36]. The inability to mount a proper immune response may also result in prolonged viral shedding, prolonging the acute complications of COVID-19 [40]. Access to standard antiviral therapies is limited due to drug-drug interactions with nirmatrelvir/ritonavir and the logistical barriers of multiday remdesivir infusions for some patients [41]. Immunobridging analyses supporting pemivibart utility in treatment—as a single-dose IV therapy with no known drug-drug interactions that mechanistically supports and precedes the native human antibody response to acute infection—could address a critical gap in care for populations who remain at high risk of severe COVID-19 outcomes.

## CONCLUSION

Following a similar approach to prevention, immunobridging was demonstrated for pemivibart for the treatment of COVID-19 suggesting substantial antiviral activity. Establishment of defined authorization or approval pathways based on such immunobridging for rapid development and approval of COVID-19 mAbs could allow sick populations and their care teams to access substantial immunologic support in the form of mAb therapy that further lowers the burden of COVID-19 disease in America. This development approach, which draws on quantitative virology and natural biological mechanisms, may be suitable to support authorization of future mAbs for COVID-19 and may have broader application in viral diseases where a rapidly evolving variant landscape necessitates accelerated novel treatments for those at risk of severe disease.

## Supplementary Material

ofag168_Supplementary_Data

## Supplementary Data

Supplementary materials are available at *Open Forum Infectious Diseases* online. Consisting of data provided by the authors to benefit the reader, the posted materials are not copyedited and are the sole responsibility of the authors, so questions or comments should be addressed to the corresponding author.
