# Supplementary material for: Immunobridging Analysis of Pemivibart for the Treatment of COVID-19: A Therapeutic Gap for the Immune-Compromised Population Remains
Source: Open Forum Infect Dis. 2026 Apr 2;13(8):ofag168. doi: 10.1093/ofid/ofag168 (PMC13501921; doi:10.1093/ofid/ofag168)
Supplement: Supplementary file 1 [file ofag168_supplementary_data.docx]

Supplementary Material

Supplementary Table 1: Relevant Dominant Variants Evaluated for Immunobridging of Pemivibart for Treatment Indication

| Variant | Pseudovirus IC_50_ (ng/mL) | Date of Peak Circulation in USA (% Frequency)* |
| --- | --- | --- |
| JN.1 | 74.6 | 02-Feb-2024 (79.1%) |
| KP.3.1.1 | 239.3 | 12-Oct-2024 (52.4%) |
| XEC | 234.7 | 18-Jan-2025 (39.7%) |
| LP.8.1 | 190.1 | 26-April-2025 (65.0%) |

IC_50_=half-maximal inhibitory concentration.

*Data obtained from published data tracker on 06-Jun-2025 using weighted model type (CDC 2025a)
